# Supplementary material for: Biochemical Analysis to Understand the Flooding Tolerance of Mutant Soybean Irradiated with Gamma Rays
Source: Int J Mol Sci. 2023 Dec 30;25(1):517. doi: 10.3390/ijms25010517 (PMC10779331; doi:10.3390/ijms25010517)
Supplement: Supplementary file 1 [file ijms-25-00517-s001.zip › Supplemental Figures.pdf]

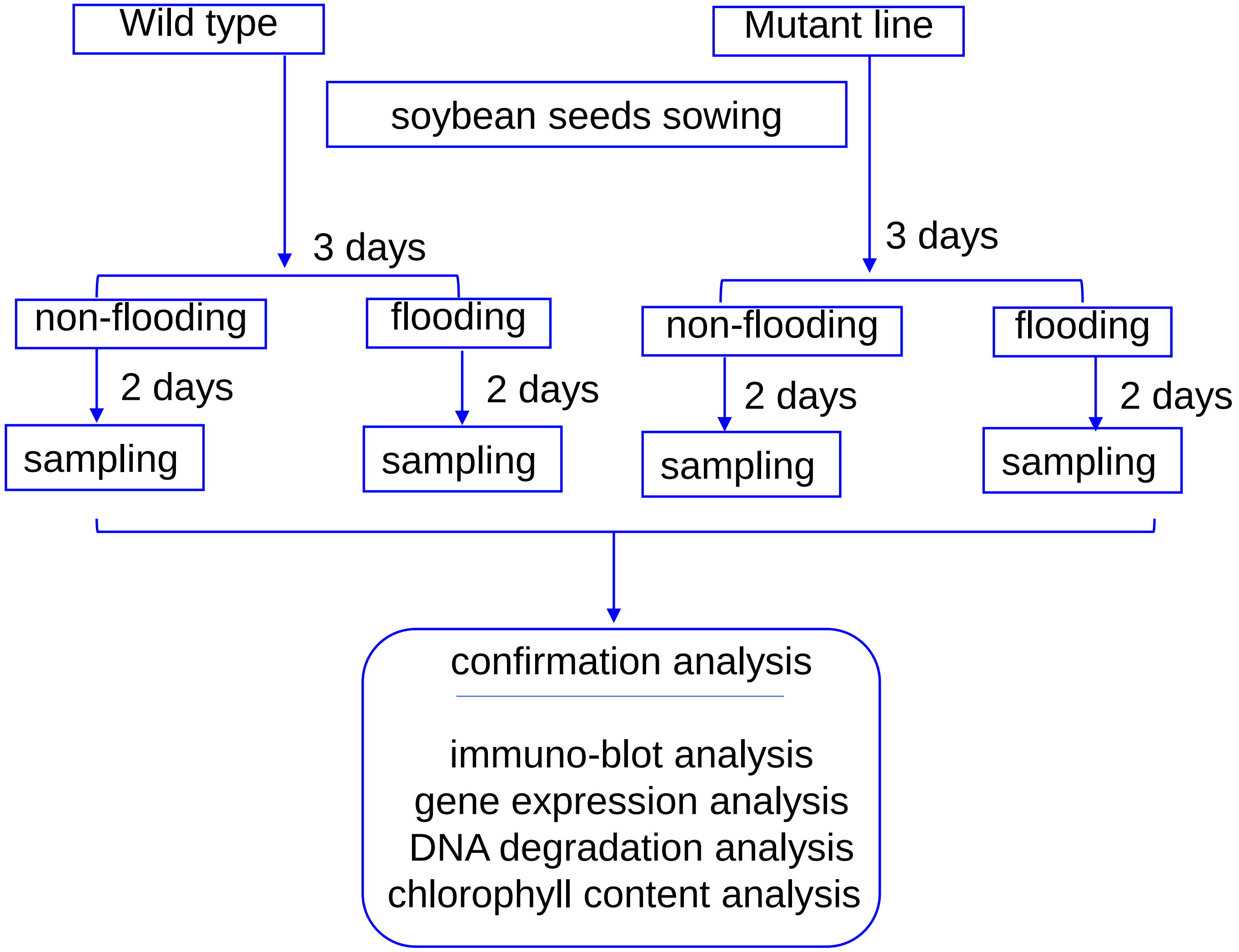

Supplemental Figure S1. Experimental design for investigation of the flooding tolerant mechanism in mutant soybean.

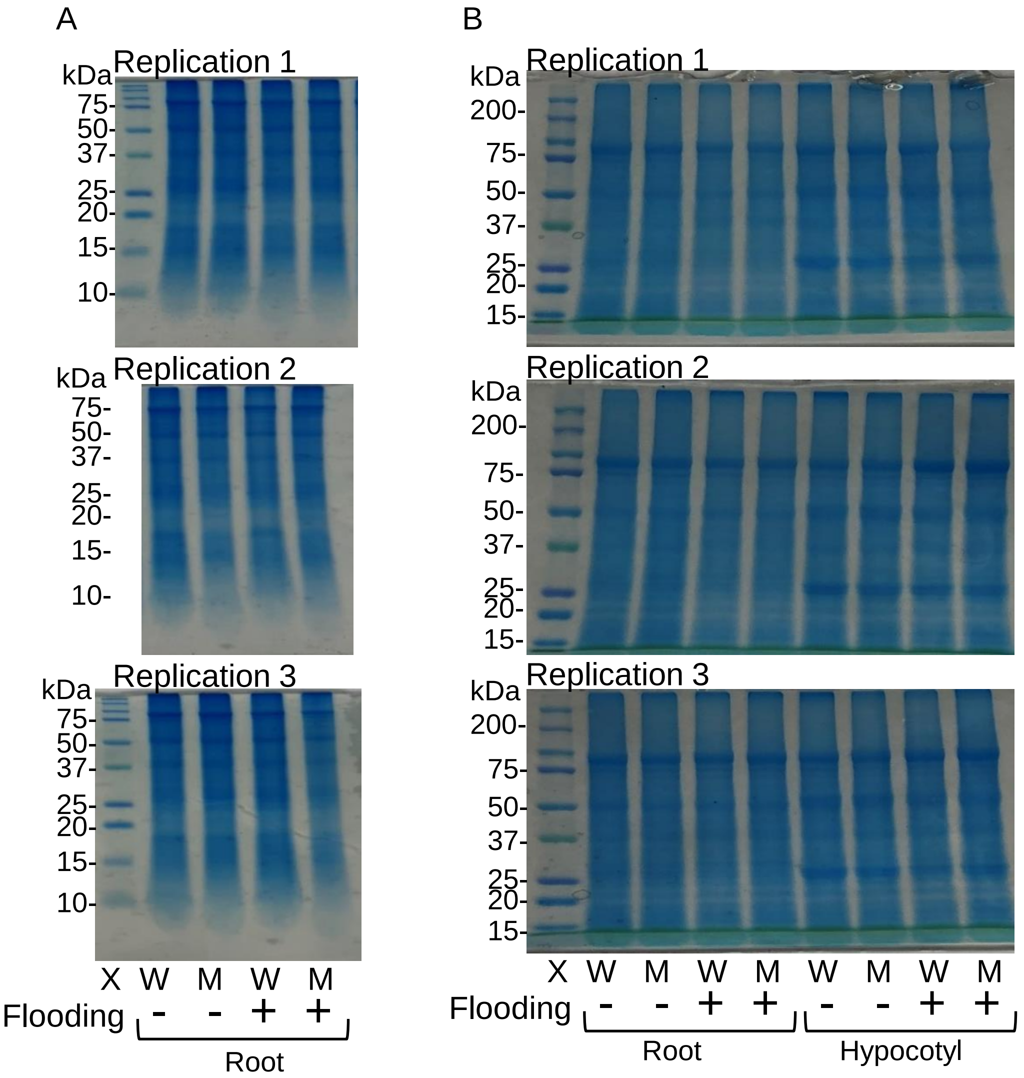

Supplemental Figure S2. The Coomassie brilliant blue staining patterns of proteins used for immuno-blot analysis. Experiments were performed with biologically triplicates for each treatments. Quantified proteins (10 µg) from root including hypocotyl were separated by electrophoresis on a 12% SDS-polyacrylamide (A) for Figure 3 or 10% SDS-polyacrylamide (B) for Figure 5. Coomassie brilliant blue staining was used as loading control. “X”, “W”, and “M” means marker proteins, protein pattern of wild type, and protein pattern of mutant line.

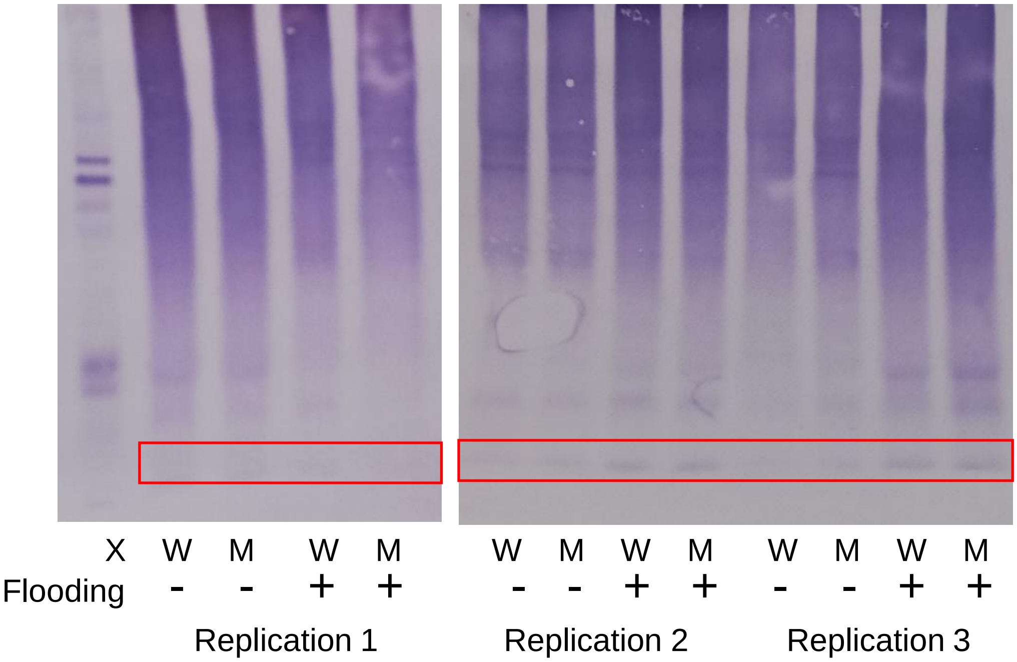

Supplemental Figure S3. Blots of the entire membrane with anti-ubiquitin antibody, which used in Figure 3. “X”, “W”, and “M” means marker proteins, wild type, and mutant line.

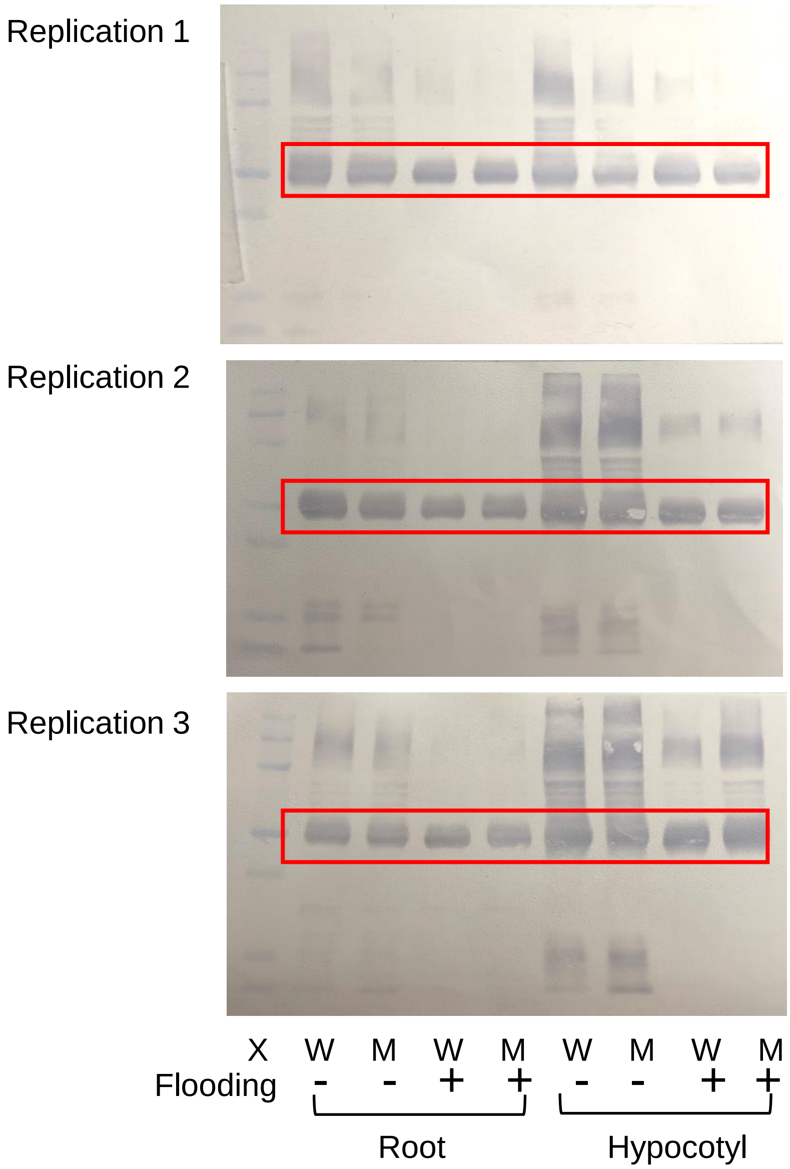

Supplemental Figure S4. Blots of the entire membrane with anti-alfa tubulin antibody, which used in Figure 5A. “X”, “W”, and “M” means marker proteins, wild type, and mutant line.

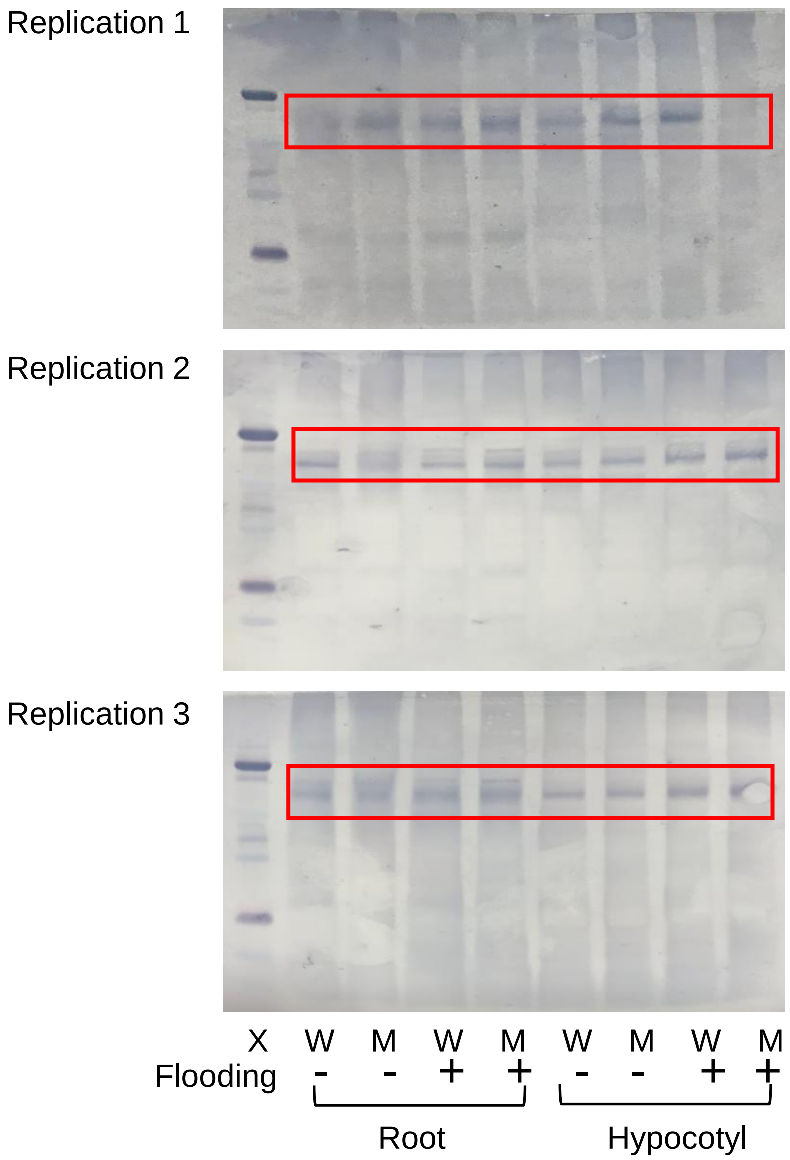

Supplemental Figure S5. Blots of the entire membrane with anti-beta tubulin antibody, which used in Figure 5B. “X”, “W”, and “M” means marker proteins, wild type, and mutant line.

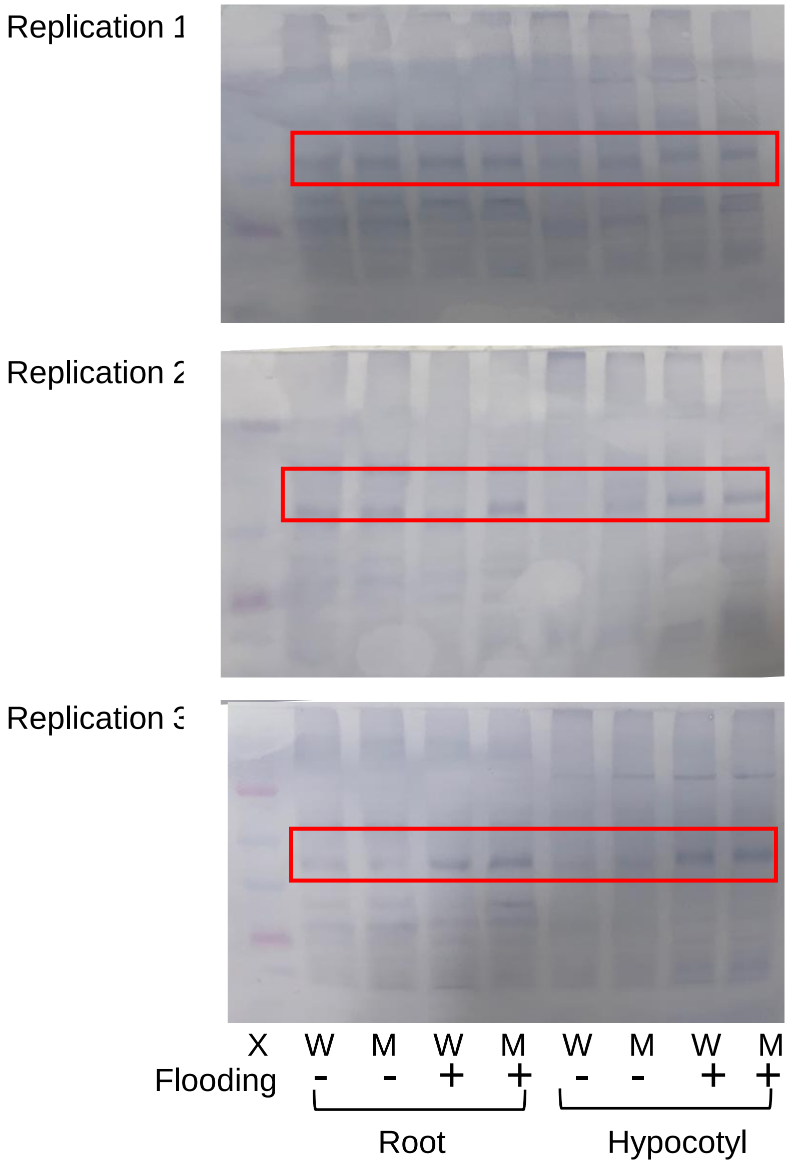

Supplemental Figure S6. Blots of the entire membrane with anti- beta tubulin antibody, which used in Figure 5C. “X”, “W”, and “M” means marker proteins, wild type, and mutant line.

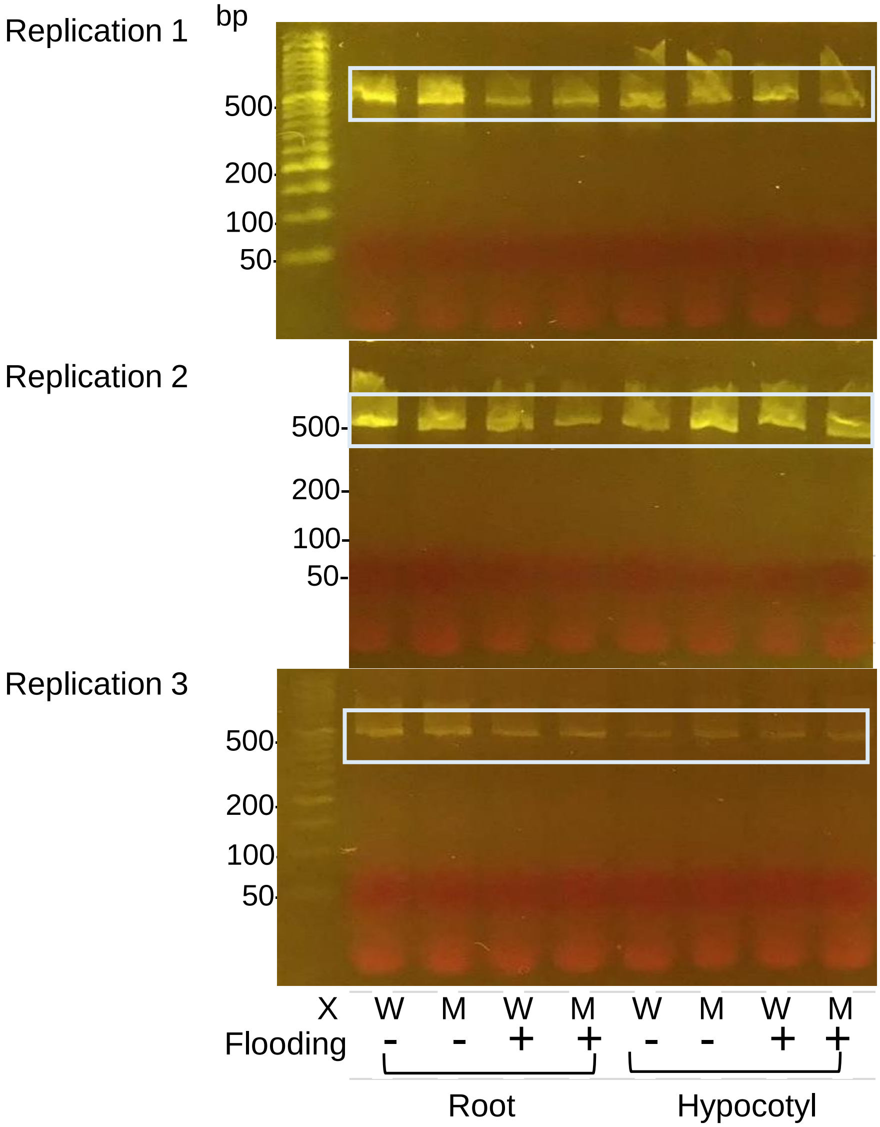

Supplemental Figure S7. Pattern of agarose gel with *60S ribosomal protein,* which used in Figure 6A. “X”, “W”, and “M” means marker proteins, wild type, and mutant line.

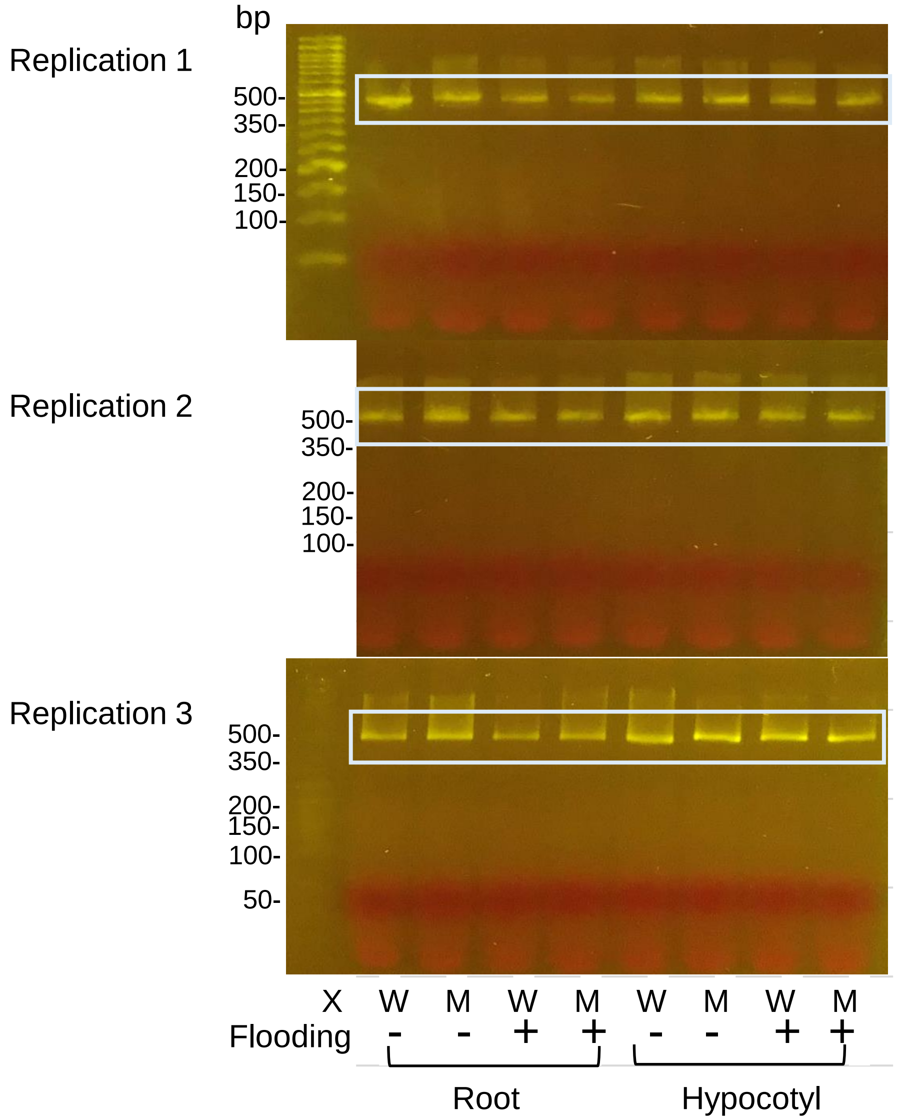

Supplemental Figure S8. Pattern of agarose gel with *RNaseH*, which used in Figure 6B. . “X”, “W”, and “M” means marker proteins, wild type, and mutant line.

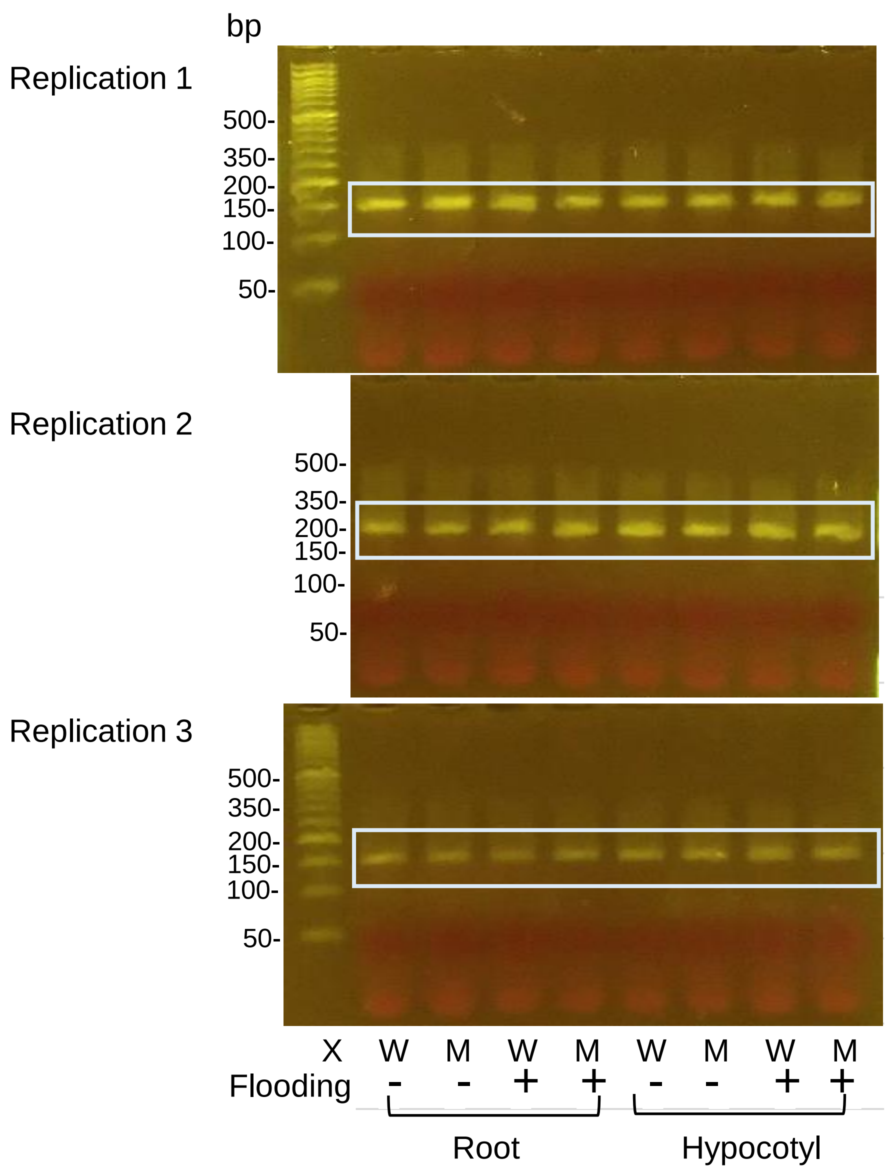

Supplemental Figure S9. Pattern of agarose gel with *18SrRNA*, which used in Figure 6. . "X", "W", and "M" means marker proteins, wild type, and mutant line.
