# Supplementary material for: Biochemical Analysis to Understand the Flooding Tolerance of Mutant Soybean Irradiated with Gamma Rays
Source: Int J Mol Sci. 2023 Dec 30;25(1):517. doi: 10.3390/ijms25010517 (PMC10779331; doi:10.3390/ijms25010517)
Supplement: Supplementary file 1 [file ijms-25-00517-s001.zip › Supplemental Table S5.pdf]

Supplemental Table S5. List of primers used for polymerase-chain reaction analysis in this research.

Name of gene	Accession	F	R
18S rRNA		TGATTAACAGGGACAGTCGG	ACGGTATCTGATCGTCTTCG
60S ribosomal protein L12-3	C6TMB5	ACTCCTTCTTCTCCGCTTCC	GTCCTTGGGATCCTTTCCAT
RNase H domain-containing protein	C6T2R5	TTAAGCCCAATCACCCTGAC	TTCCCAGAAGATCGACAACC
