# Supplementary figures and images for: Biochemical Analysis to Understand the Flooding Tolerance of Mutant Soybean Irradiated with Gamma Rays
Source: Int J Mol Sci. 2023 Dec 30;25(1):517. doi: 10.3390/ijms25010517 (PMC10779331; doi:10.3390/ijms25010517)

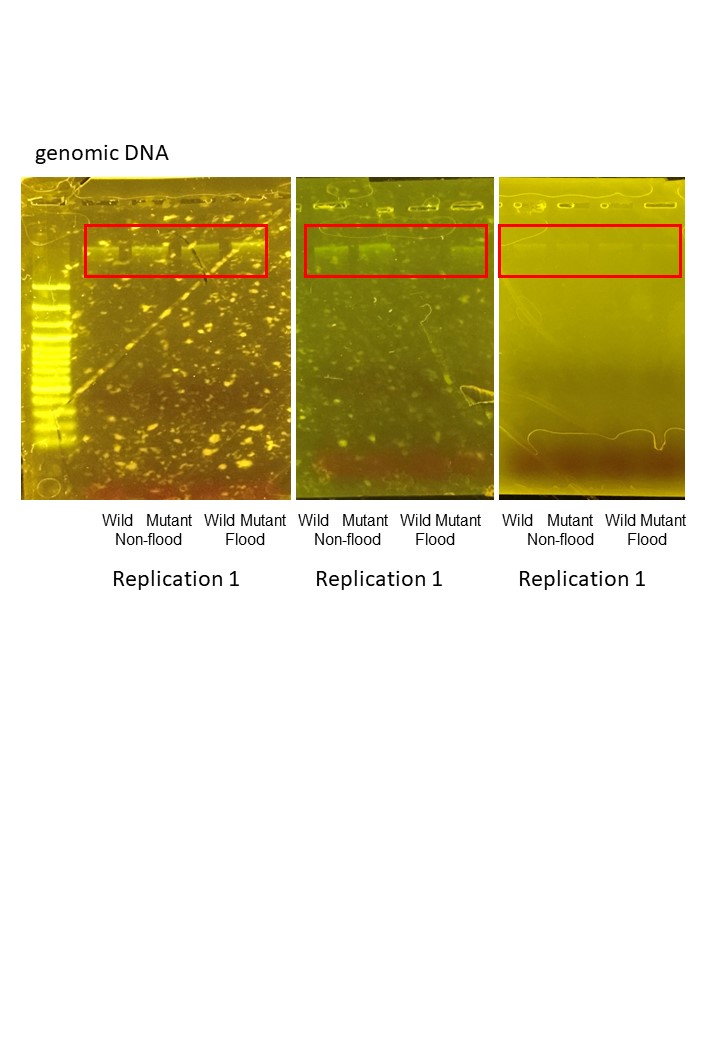

Supplement: Supplementary file 1 [file ijms-25-00517-s001.zip › Additional Figure for Figure 4 (genomic DNA).jpg]
